# Considering Theory-Based Gamification in the Co-Design and Development of a Virtual Reality Cognitive Remediation Intervention for Depression (bWell-D): Mixed Methods Study

**DOI:** 10.2196/59514

**Published:** 2025-03-31

**Authors:** Mark Hewko, Vincent Gagnon Shaigetz, Michael S Smith, Elicia Kohlenberg, Pooria Ahmadi, Maria Elena Hernandez Hernandez, Catherine Proulx, Anne Cabral, Melanie Segado, Trisha Chakrabarty, Nusrat Choudhury

**Affiliations:** 1 National Research Council Winnipeg, MB Canada; 2 National Research Council Boucherville, QC Canada; 3 Department of Psychiatry Faculty of Medicine University of British Columbia Vancouver, BC Canada

**Keywords:** virtual reality, clinical psychology, cognitive assessment, neuropsychology, mental health, cognitive training, cognitive remediation, cognitive rehabilitation, digital therapeutics

## Abstract

**Background:**

In collaboration with clinical domain experts, we developed a prototype of immersive virtual reality (VR) cognitive remediation for major depressive disorder (bWell-D). In the development of a new digital intervention, there is a need to determine the effective components and clinical relevance using systematic methodologies. From an implementation perspective, the effectiveness of digital intervention delivery is challenged by low uptake and high noncompliance rates. Gamification may play a role in addressing this as it can boost adherence. However, careful consideration is required in its application to promote user motivation intrinsically.

**Objective:**

We aimed to address these challenges through an iterative process for development that involves co-design for developing content as well as in the application of gamification while also taking into consideration behavior change theories. This effort followed the methodological framework guidelines outlined by an international working group for development of VR therapies.

**Methods:**

In previously reported work, we collected qualitative data from patients and care providers to understand end-user perceptions on the use of VR technologies for cognitive remediation, reveal insights on the drivers for behavior change, and obtain suggestions for changes specific to the VR program. In this study, we translated these findings into concrete representative software functionalities or features and evaluated them against behavioral theories to characterize gamification elements in terms of factors that drive behavior change and intrinsic engagement, which is of particular importance in the context of cognitive remediation. The implemented changes were formally evaluated through user trials.

**Results:**

The results indicated that feedback from end users centered on using gamification to add *artificial challenges*, *personalization* and *customization* options, and *artificial assistance* while focusing on *capability* as the behavior change driver. It was also found that, in terms of promoting intrinsic engagement, the need to meet *competence* was most frequently raised. In user trials, bWell-D was well tolerated, and preliminary results suggested an increase in user experience ratings with high engagement reported throughout a 4-week training program.

**Conclusions:**

In this paper, we present a process for the application of gamification that includes characterizing what was applied in a standardized way and identifying the underlying mechanisms that are targeted. Typical gamification elements such as *points* and *scoring* and *rewards* and *prizes* target *motivation* in an extrinsic fashion. In this work, it was found that modifications suggested by end users resulted in the inclusion of gamification elements less commonly observed and that tend to focus more on individual ability. It was found that the incorporation of end-user feedback can lead to the application of gamification in broader ways, with the identification of elements that are potentially better suited for mental health domains.

## Introduction

### Background

Major depressive disorder (MDD) is the leading cause of disability worldwide [1]. Current treatment regimens, while effective in improving core symptoms of MDD, fall short in addressing MDD-related disability [2-4]. Cognitive dysfunction—encompassing deficits in memory, processing speed, attention, and executive functioning—has emerged as an important determinant of functional outcomes in MDD [5-7]. Advancing cognitive-targeting therapies such as cognitive remediation has accordingly become a critical treatment priority. Cognitive remediation is a behavioral-based therapy aimed at enhancing neurocognitive skills, typically through a combination of “drill-and-practice” cognitive games and implementation of compensatory strategies [8]. Traditional cognitive remediation programs delivered in paper-and-pencil or computerized formats have demonstrated robust improvements in both cognition and real-world functioning in schizophrenia [9], attention-deficit/hyperactivity disorder, and autism spectrum disorder [10]. In MDD, results to date have been equivocal. Although cognitive remediation in patients with MDD appears to improve performance in select cognitive domains, this has not translated to appreciable improvements in real-world functioning [11,12].

Immersive virtual reality (VR) has emerged as a promising modality to enhance the real-world effectiveness of cognitive remediation. Such technologies allow for almost complete sensory immersion with extensive design possibilities. By creating simulations that engage users via multiple senses (sight, hearing, and touch) while permitting natural movement, immersive VR creates a sense of presence that can elicit a genuine response in the individual. The use of VR environments also permits tight experimental control, which makes it feasible to measure and study everyday functioning that can be otherwise prohibitive in real-world settings. The ability of VR to deliver and control stimuli while capturing responses with high fidelity during an exercise provides a controlled and repeatable tool that is unavailable in traditional testing or remediation methods and makes it ideal for assessing and training cognitive skills in the performance of simulated real-life tasks. There has been growing interest in the use of VR for both assessment and rehabilitation from the research and clinical neuropsychology communities in recent years [13,14]. The feasibility of using VR for cognitive assessment and care has been demonstrated across various cognitive domains, such as attention [15-21], executive functions [22-25], memory [26-31], and spatial abilities [32-34].

The National Research Council Canada (NRC) has developed bWell, a VR-based immersive platform for cognitive assessment and remediation [35]. bWell is a foundational platform featuring configurable exercises and designed as a broadly applicable toolkit targeting general aspects of cognition that are commonly impacted across many disorders. A video sample of bWell’s interface and laboratory exercise are presented in Multimedia Appendix 1. Preliminary acceptability studies in healthy individuals have indicated that the platform is enjoyable, engaging, and well tolerated [36,37]. These studies have also shown that using immersive VR for clinical applications not only is technically feasible but also has advantages over traditional 2D equivalents. The first study showed that subjective reports of engagement when performing a task in bWell (an immersive environment) were greater than those when performing the same task on a tablet (a nonimmersive environment). The second study showed that the use of more complex types of immersion (eg, the user was not static) did not increase subjective reports of cybersickness during a visual attention task and provided the required support for testing in clinical populations.

bWell is currently being incorporated into clinical settings using an interdisciplinary approach successfully developed by the NRC [38-41]. This method, which has proven effective in creating surgical simulation platforms, most notably for neurosurgery (eg, NeuroTouch, distributed worldwide as NeuroVR by CAE Healthcare), ensures a comprehensive and collaborative framework for implementation. Specifically, the team established long-term collaborative research agreements with 5 world-renowned clinical sites across Canada and 1 in Japan—the NRC’s cognitive care network (CCN). The CCN consists of an early adopter group that provided domain-specific perspectives as well as feedback on iterative deliveries of the platform as bWell development progressed. The CCN is currently launching several studies investigating the content of bWell exercises, as well as its use, specific to target populations.

In one of these collaborations, with the University of British Columbia, we customized bWell to deficits observed in depression as bWell-D, a prototype of immersive VR cognitive remediation for MDD. bWell-D targets cognitive and real-world challenges germane to MDD depression [6]. It uses previously identified common components of successful cognitive remediation in psychiatric populations [42]. These include (1) *errorless learning*, wherein participants are provided support in learning all task components, with supports gradually removed as skills and confidence increase; and (2) *adaptivity*, where tasks become increasingly complex to match the participants’ growing competence.

In the development of a new digital technology, there is a need to determine the effective components and clinical relevance using systematic methodologies. A working group of international experts has published the first consensus guidelines for best practices in the design and testing of VR clinical applications [43]. In these guidelines, incorporation of qualitative feedback from multiple end users is identified as a critical component of VR program content development. In addition, the Medical Research Council (United Kingdom), in a guidance document on the development of complex interventions to improve health and health care [44], recommends including prevailing theories and frameworks in a given domain to anchor the feedback obtained.

From an implementation perspective, the effectiveness of digital intervention delivery is challenged by low uptake and high noncompliance rates, which gamification may play a role in addressing. A systematic study of technology use in the treatment of mental health disorders found that, specific to depression, gamification was being used as an approach aiming to improve adherence and engagement [45]. The application of gamification to mental health domains is still a new research field with limited studies [46] that tend to be heterogeneous [47]. The results are inconsistent; for example, it was observed that its application to web-based interventions did not lead to a significant increase in adherence [48]. There is also a lack of comparison between gamified and nongamified interventions, which makes it difficult to evaluate the impact of such features on adherence and engagement. Finally, it has been suggested that gamification might be used without fully understanding the behavioral theories or mechanisms behind it [46,49]. For instance, adding too many gamification elements that rely on external rewards can overwhelm the user with unnecessary cognitive demands, potentially invalidating the task [47].

### Objectives

Using behavioral theory as a framework for understanding likely processes for change induced by an intervention is hypothesized to enhance its effectiveness [40,50]. The capability, opportunity, and motivation–behavior (COM-B) model [51] is one such theory that provides valuable insights. It is widely used and identifies *capability*, *motivation*, and *opportunity* as the 3 essential elements for all behavior. Effecting behavior change, such as improving compliance with a digital intervention, requires addressing at least one of these drivers of behavior. Another relevant behavioral theory is self-determination theory, which delves deeper into the aspects of motivation. This theory posits that people are intrinsically motivated to grow and change when their needs for *autonomy*, *competence*, and *connection* are met [52].

## Methods

### Overview

In this work, we aimed to address these challenges through an iterative process for the development of bWell-D that involves co-design for the development of content and in the application of gamification using behavior change theories as a framework.

Following best practice guidelines, qualitative data were collected from patients and care providers to understand end-user perceptions on the use of VR technologies for cognitive remediation and obtain suggestions for changes specific to the bWell-D program. This qualitative study phase was reported in a previous paper from the authors [53]. Briefly, participants comprising individuals with lived experience of depression (n=15) and clinicians (n=12) were interviewed, and the results were transcribed and coded followed by thematic analysis. The demographic and clinical characteristics of these end users are described in our previous paper. Interviewees were asked questions to probe drivers of behavior change related to the use of VR (eg, perceptions and experiences with VR, desired outcomes from a workplace cognitive and functional remediation program, and perceived barriers). Participants were also asked for feedback specific to the bWell-D program itself, for which they were shown previously recorded video samples of the tasks and probed for potential relevance to daily life and how to improve the tasks to enhance engagement and real-world applicability. Emerging themes from patients and clinicians were identified and ranked based on prevalence.

The focus of this paper was to structure these previously collected qualitative data into a theoretically grounded model to guide software and functionality development for the next iterations of bWell-D. Specifically, we aimed to use these findings to implement features for the next iterations of bWell-D specific to its use in cognitive remediation while promoting the required motivation, engagement, and ease of use or access to obtain favorable outcomes. Following best practice guidelines for human-centered VR content development [43], the themes suggested by end users to improve the bWell-D program were discussed among the research team and translated into representative software functionalities during team brainstorming sessions. It was found that the functionalities that could be implemented could also be described in many ways. In an effort to standardize the categorization, all functionalities were encoded against gamification elements based on the taxonomy by Cheng et al [49], who describe 18 elements used in digital technologies for 17 mental health and well-being domains, including depressive disorders.

The gamification elements were then evaluated against behavioral theories to characterize each element in terms of (1) factors that drive behavior change and (2) potential to intrinsically promote user engagement. The COM-B factors and psychological needs of self-determination are presented in Textbox 1 [51,52] to provide a clearer definition.

Taking into consideration the factors identified from behavioral theories (eg, opportunity), the ideation included both updating the concepts for each bWell-D exercise as well as the overall delivery and use of the bWell-D program itself. The modifications were assigned priority for implementation based on (1) how frequently these changes were suggested by end users and (2) whether these changes were feasible for development in a VR environment.

**Figure 1:**
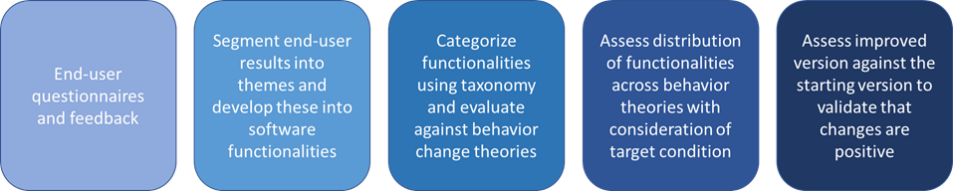
Iterative software development process using end-user feedback, thematic segmentation of feedback, software functionality development, taxonomy assignment, assessment using selected behavior change theories, distribution review, and functionality validation for improvement.

The steps of the methodology used are summarized in Figure 1. End-user feedback was thematically grouped and developed into software functionalities. These functionalities were categorized using a taxonomy for digital technologies used in mental health and well-being applications. Furthermore, the functionalities were assessed using selected behavior change theories to view the distribution of needs identified by end users for platform improvement. The last step involves validating the implemented software functionalities through an iterative development process that gathers new feedback from end users for further refinement. Multiple rounds of iteration will help create a comprehensive overview of the functionalities that end users want and refine these implementations to ensure that they effectively meet users’ needs. This overview will highlight the range of engagement methods preferred by users organized according to a taxonomy of gamification elements for digital health applications. In addition, selected behavior change theories will provide insights into what motivates users to engage with and adhere to treatments.

Finally, in this study, new participants were recruited for a pilot feasibility trial with a healthy population. With the aim of evaluating the impact of the gamification elements, participants trialed the original version for 40 minutes, after which they were given the option to either try out the expanded features of the bWell-D platform for up to 15 minutes or embark on a training program for 8 sessions (occurring 2 times a week for 4 weeks). Feedback regarding tolerability and cybersickness was obtained using the Simulator Sickness Questionnaire (SSQ) [54] before and after each session. User experience feedback was obtained using the User Engagement Scale (UES) [55] and Game User Experience Satisfaction Scale (GUESS) [56] after the initial assessment session and after the first, fourth, and last training sessions. Ad hoc comments made by the participants during the trials were also noted. For participants who chose to try out the expanded feature without attending the training sessions, feedback was obtained via a semistructured interview while performing the exercises.

**Categorization in terms of factors that drive behavior change and potential to intrinsically promote engagement.**

**Categorization for effective behavior change**

- The capability, opportunity, and motivation–behavior model posits that there are 3 drivers for behavior change. (1) *Capability* is the ability to engage in an activity; (2) *opportunity* is factors that lie outside the individual that can make behavior possible, prompt it, or present a barrier to it; and (3) *motivation* is the brain processes that energize and direct behavior.

**Categorization for intrinsic engagement**

- According to self-determination theory, 3 needs must be met to achieve psychological (inner) growth. (1) *Autonomy* is when a person feels in control of their own behaviors and that their action will result in real change, it plays a major part in helping them feel self-determined; (2) *competence* is when a person feels equipped with the skills needed, they are more likely to take action; (3) *connection or relatedness* is a sense of belonging and attachment to others promotes the actualization of inherent potential. Social environments supporting these needs enhance one’s internal motivation.

### Ethical Considerations

The research and studies were reviewed and approved by the institutional research ethics board at the University of British Columbia (H21-00028 and H20-00746) and the NRC (2020-122). Informed consent was obtained before the start of the study. Data were deidentified before any analysis. Participants were compensated for taking part with CAD $100 (US $70).

## Results

### Overview

Results from the previously published qualitative study [53] showed that, overall, patients and clinicians considered bWell-D to be interesting, acceptable, and potentially feasible and provided suggestions to enhance its applicability. Five main themes were identified from the patient interviews: (1) patients would prefer to undergo VR treatment at home, (2) the VR treatment should emulate real-life settings, (3) the VR intervention needs to be multisensory, (4) the VR treatment should be challenging, and (5) the VR treatment should be engaging. Three main themes were identified from the clinician interviews: (1) the VR treatment should be multi-domain, (2) the VR tasks should become more challenging over time, and (3) remote treatment would be more convenient for patients. In addition, end users suggested having a user-friendly interface and tutorials to have an opportunity to practice the tasks, which they considered especially important for at-home treatment. Participants also recommended including an additional VR activity that specifically targets their mood.

In this study, these themes were evaluated against the identified behavioral theories, and it was found that the themes encompassed all 3 COM-B drivers for behavior change. Regarding *motivation*, VR is seen as a potential, interesting, but underexplored treatment option for remediating cognitive deficits in depression. Many suggestions for bWell were related to better harnessing the affordances of VR to deliver multisensorial and multimodal exercises. Regarding *capability*, VR was seen as “the second-best thing to real life” as it allows for immersion in highly realistic settings. Some skepticism about effectiveness in the short and long term was also expressed, especially when the purpose and real-world applications of practiced skills were not made explicit. Regarding *motivation*, the gamified experience of VR adds fun, engaging, and immersive elements to the treatment. There is a need for a good balance in challenge (motivating but not too hard) for continued engagement. Regarding *opportunity*, there is a need for inclusivity and customizability in treatment delivery. Participants believed that the option for home-based treatment provided a sense of comfort and control. However, it was not the preference for everyone. Specifically, patients with limited space or suboptimal conditions for treatment at home would prefer having the option to receive treatment at a clinic if necessary. Concerns about equipment accessibility were also highlighted.

In terms of feedback specific to bWell-D, the main areas of improvement suggested by both clinicians and patients centered on (1) *motivation*—taking greater advantage of VR multisensorial affordances, (2) *capability*—the inclusion of more realistic VR environments, and (3) *motivation*—providing sufficient challenge for fun and engaging tasks.

It was observed that patient and clinician themes overlapped on several common topics, and thus, they were combined. Similarly, the feedback specific to bWell was related to that for the general use of VR. However, while the suggestions specific to bWell addressed 2 of the drivers—*motivation* and *capability*—*opportunity* was not captured. In an aim to capture all drivers for behavior change as well as all the major suggestions from end users, the following overall themes emerged as priorities for the development of bWell-D: (1) provide sufficient *challenge* over time for fun and engaging tasks (patient themes 4 and 5 and clinician theme 2), (2) take greater advantage of the potential of VR to create tasks that are more *multi-domain* and *multisensorial* (visual, auditory, and tactile stimuli; patient theme 3 and clinician theme 1), (3) include more *representative* VR elements (to reflect more realistic VR environments; patient theme 2), (4) increase *autonomy*, opportunity, or enablement with remote or *in-home* use (patient theme 1 and clinician theme 3), and (5) provide *mood-focused* features and tasks (participant recommendation).

The gamification elements for each exercise, including relevant original elements and the newly implemented modifications based on qualitative feedback, are summarized in the upcoming sections. Each element is classified according to gamification taxonomy and characterized according to the drivers of behavior change from the COM-B framework it relates to (*motivation*, *capability*, or *opportunity*), as well as the need from self-determination theory that it most addresses (*autonomy*, *competence*, or *connection*)*.* Each element is also associated with overall themes summarized from the qualitative analysis.

### General Interface, Tutorial, and bWell-D Administration

**Figure 2:**
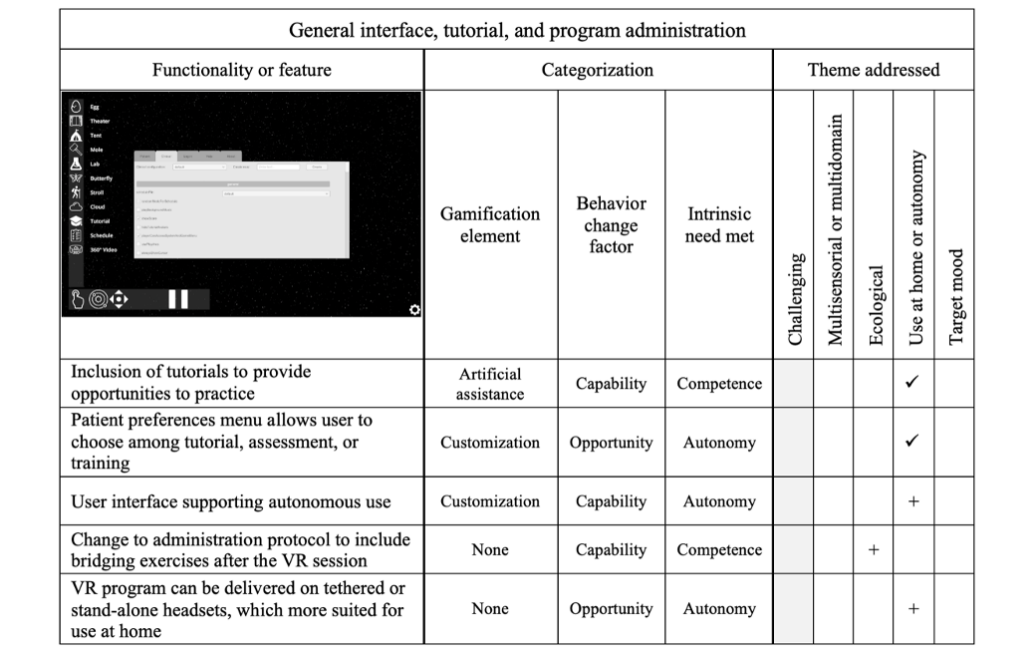
General interface, tutorial, and program administration, including modifications made after end-user feedback. Checkmarks (✓) denote existing elements and plus signs (+) denote new elements that were added in the updated version of bWell-D. Gray shading indicates that the theme does not apply. VR: virtual reality.

The general modifications to bWell-D are described in this section, and Figure 2 provides a summary of the categorization.

bWell-D has 3 distinct modes to optimize user engagement and outcomes. (1) Tutorial mode is for initiation with the virtual environment, providing a comprehensive walkthrough of the tasks to be undertaken. It ensures that users are comfortable and well prepared to navigate the tasks effectively. (2) Assessment mode serves as a diagnostic tool to evaluate the users’ abilities by monitoring their performance at a consistent level of difficulty. The primary objective is to pinpoint the users’ unique requirements and areas where they may need additional support or intervention. (3) Training mode is tailored to foster user growth. The training mode dynamically adjusts the difficulty level in response to individual performance. This personalized approach ensures that the tasks remain challenging yet achievable, promoting continuous skill development. Users receive instantaneous feedback on progress via level or score change notifications.

The modes can be configured and customized via a clinical interface as bWell-D is intended for administration by a facilitator.

On the basis of the emerging theme of increased opportunity with at-home use, a new user interface was implemented to support autonomous use. As a result, bWell-D’s hardware-agnostic core has now been leveraged to integrate stand-alone headsets such as the PICO Neo3 Eye and the Oculus Quest 2. Such headsets have come a long way, from clunky, tethered devices to performant and comfortable stand-alone units aimed at the mass market that enable seamless onboarding of nontechnical users new to VR.

Existing features of bWell did not include the emerging theme of representative real-world elements (Figure 2). With the aim to make the purpose and real-world applications of practiced skills more explicit, bWell-D administration now includes “bridge exercises” as a supplement to the VR program. These exercises consist of asking participants to reflect on how they think the VR tasks will apply to their personal contexts to help them understand how practiced skills could be related to their real life.

### Egg Scene

**Figure 3:**
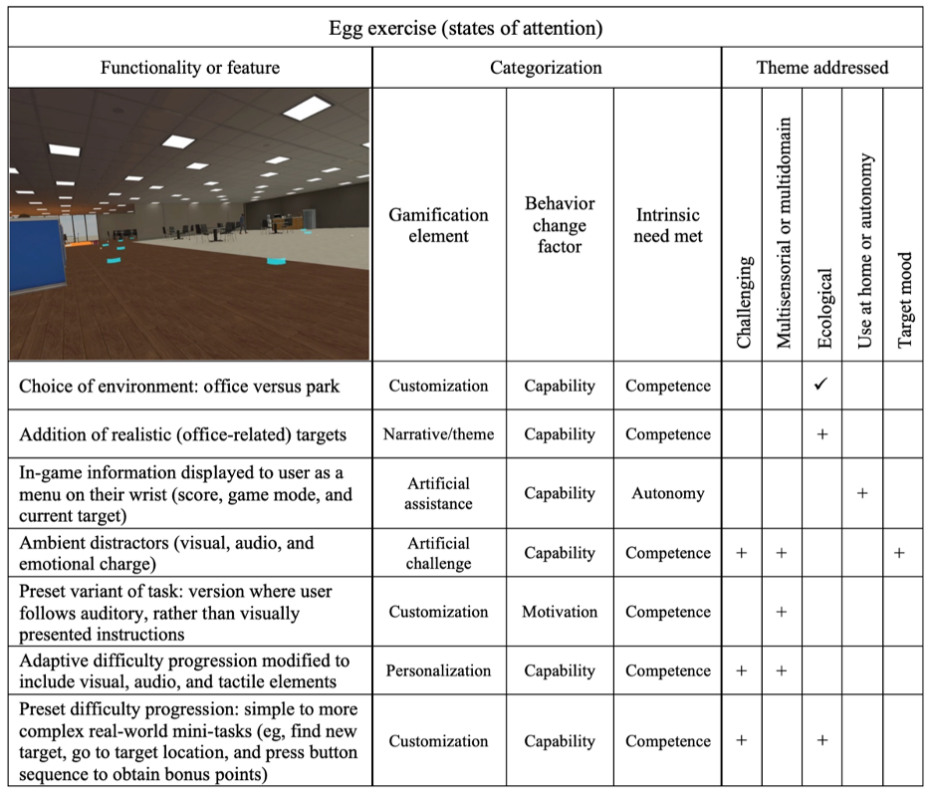
Egg exercise (targeting states of attention), including modifications made after end-user feedback. Checkmarks (✓) denote existing elements and plus signs (+) denote new elements that were added in the updated version of bWell-D.

The egg scene takes place in an office work environment. The user is tasked with scanning the environment for eggs, drawing on visual processing and sustained attention. They are then required to direct and hold their gaze on a located egg long enough to make it hatch. Modifications to the scene and a summary of their categorization are presented in Figure 3, where it can be observed that only the theme of representative real-world elements has been addressed in the existing features (ie, creation of a simulated real-world environment).

In the feedback obtained, end users recommended including more real-world VR elements. To address this, the egg target was changed to new, more context-appropriate ones for the office-themed virtual environment. These included staplers, legal pads, and coffee mugs.

An in-game user interface was added to the player’s wrist during the egg scene to display important information, including the current score, target object, and target location. This type of interface takes advantage of the user’s natural arm movements, allowing them to easily access information or controls by looking at or interacting with their wrist, much like checking a smartwatch. It is designed to be intuitive and accessible without interrupting the immersive experience. This *artificial assistance* element addressed the need for *autonomy.*

Ambient, multisensorial distractors were added as options to the scene. Visual distractors include videos playing on computer screens, coworkers walking around the office, and fake eggs that resemble the real target eggs. Audio distractors include phone rings, coworkers talking, and a buzzing sound that emanates from randomized locations within the office. These distractors add *artificial* *challenge* as well as addressing the mood theme by introducing an element of hot cognition.

A *customization* option was added in which users follow auditory rather than the usual visual instructions. This addresses the user’s *motivation*, providing an alternative sensorial interaction pathway to engage with the user.

An adaptive difficulty progression including multisensorial stimuli was developed for the scene. To address the user’s *capability*, difficulty adjusts based on user performance, making this a *personalization* element. Difficulty adjustment includes adjusting the number of targets, the reaction delay allowed to obtain bonus points, the time it takes to hatch an egg, and the number and frequency of audio distractors. The user’s performance is assessed based on how they respond to multiple stimuli: a visual (egg turns green), tactile (controller vibrates), or audio (clock ticking sound clip) stimulus.

The last modification to the scene was the addition of a preset difficulty progression. The user is given tasks, starting off simple at lower levels and becoming increasingly complex at higher levels. These tasks include finding a new target, going to a target location, and pressing a button sequence to obtain bonus points. Because these levels are chosen by the user or clinician rather than being adaptive, they are considered a *customization* element.

### Laboratory Scene

In the laboratory scene, the user must complete 2 recipes simultaneously by pouring the correct ingredients at the correct times into 2 different tubs. The scene challenges the user’s executive function and attention across several dimensions, including divided, selective, and sustained attention. A supervisor avatar is present in the scene and adds *artificial* *assistance* by giving auditory feedback on the user’s performance and advice on how to improve.

**Figure 4:**
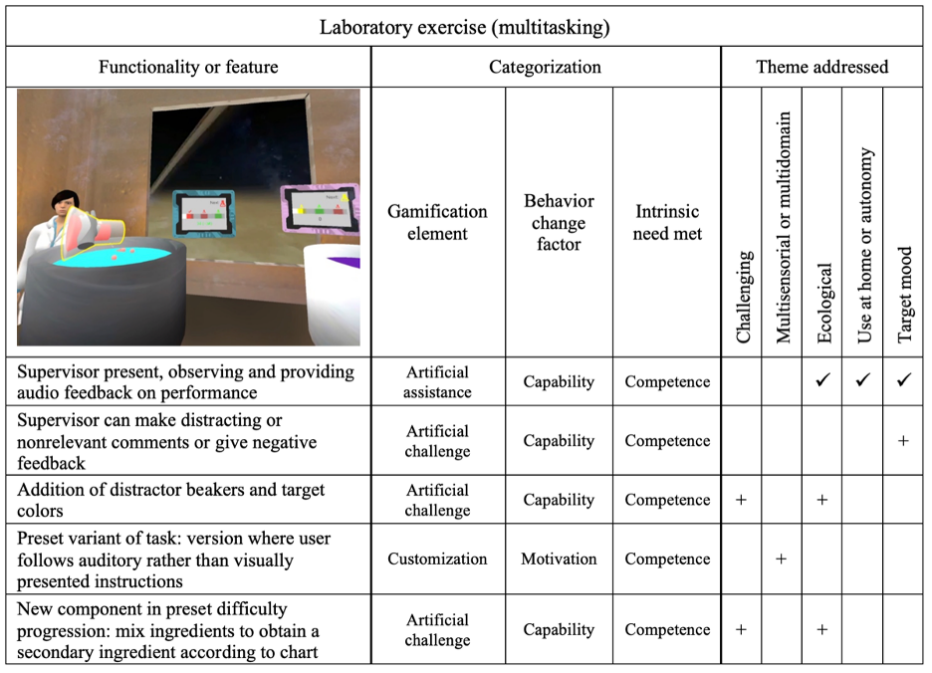
Laboratory exercise (targeting multitasking), including modifications made after end-user feedback. Checkmarks (✓) denote existing elements and plus signs (+) denote new elements that were added in the updated version of bWell-D.

Modifications to the scene and a summary of their categorization are presented in Figure 4. On the basis of the identified theme of added challenge and the inclusion of more representative elements, a *customization* option was added in which the supervisor avatar gives the user auditory instructions rather than having the recipes visually presented on tablets. An aspect of hot cognition was also added by modifying the avatar behavior so that it can make distracting or irrelevant comments to the user.

In total, 2 modifications were made to the scene explicitly to add *artificial* *challenge*. The first one simply added 3 new nonrelevant ingredient bottles as distractions while the user attempts to complete the recipes. The second modification was the introduction of new secondary ingredients to recipes. The user must first create the secondary ingredient by combining 2 primary ingredients in a beaker before pouring the beaker in the tub, adding complexity to the task. A chart showing possible ingredient combinations was added to the scene.

### Mole Scene

The mole scene is a *Whac-A-Mole* variation in which the user has a colored hammer in each hand with which they are to hit matching colored cylinders (moles) that pop up in front of them. Hammer colors change unexpectedly from time to time. Hitting valid moles exercises the user’s impulse control with selective attention, visual processing, and executive functioning.

A score streak animation (visual reward) is used to provide *level* and *progress* *feedback*, serving to *motivate* the user to maintain a fast pace as the difficulty increases. This occurs when multiple moles are correctly hit in sequence. In addition, to enable investigations of potential bias in MDD, the moles were modified to have a simple facial expression, either a smile or a frown. The program checks whether the particular facial expression influences the accuracy of the hits. In one mode, valid moles (ones with a color matching a hammer) are assigned a smiling expression, and invalid moles are frowning. In another mode, the expressions are randomized.

**Figure 5:**
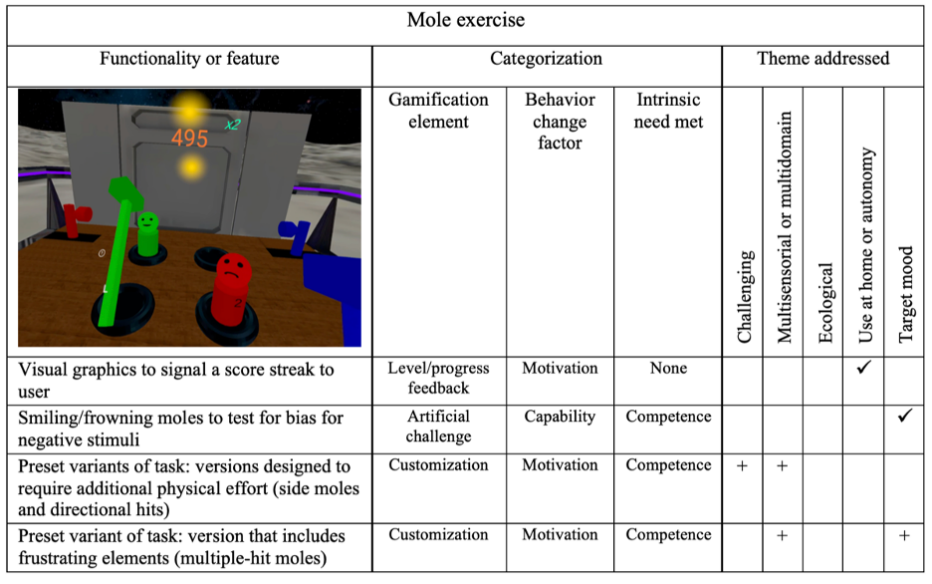
Mole exercise (targeting response inhibition and cognitive control, including modifications made after end-user feedback). Checkmarks (✓) denote existing elements and plus signs (+) denote new elements that were added in the updated version of bWell-D.

Modifications to the scene and a summary of their categorization are presented in Figure 5. From Figure 5, it can be observed that the existing features did not include the emerging theme of more multi-domain and multisensorial elements. The changes focused on exercise variants to address other domains. The multiple-hit mole *customization* variant was added to target mood. The multiple-hit mole is a variant taken from the bWell core battery of exercises designed to frustrate the user by requiring repeated hits for the mole to go down. In total, 2 other bWell core *customization* variants were added to the mole scene. The side moles and directional hit variants require additional physical effort or dexterity. Side moles appear to the side of the main table, requiring the user to turn their head and extend their arms further to hit them. In the directional hit variant, moles must be hit on the top of their head to score points. This is more challenging compared to the usual case in which moles can be hit from any angle and still score points. It was hoped that the additional physical engagement required by these variations would increase *motivation* for users to continue using the program.

### Theater Scene

The theater scene is a matching task for visual and short-term memory. The user is presented with a set of target shapes for a short period and asked to remember it. The target shapes are then hidden from view. After a set time, random shapes start falling into view, and the user must reproduce the initial series of shapes. The scene was modeled after a university classroom, with the teacher’s desk in front and the students’ desks laid out in a semicircle in front of it. This setting provides an ecological environment for the sample population, which were recruited on a university campus. The user is placed in front of the teacher, who is seated at their desk. A few student avatars who may be enabled or disabled are seated in the classroom behind the user. This arrangement was intended to be somewhat daunting. For additional distraction, the teacher in front can create noise by taking down notes, and the student avatars can make loud whispering noises. The arrangement and the distractions add a level of *artificial* *challenge* for the user as well as hot cognition.

Further *artificial* *challenge* was added by including distracting shapes that are not part of the correct answer. The pool size of distracting shapes was increased to further enhance the challenge.

**Figure 6:**
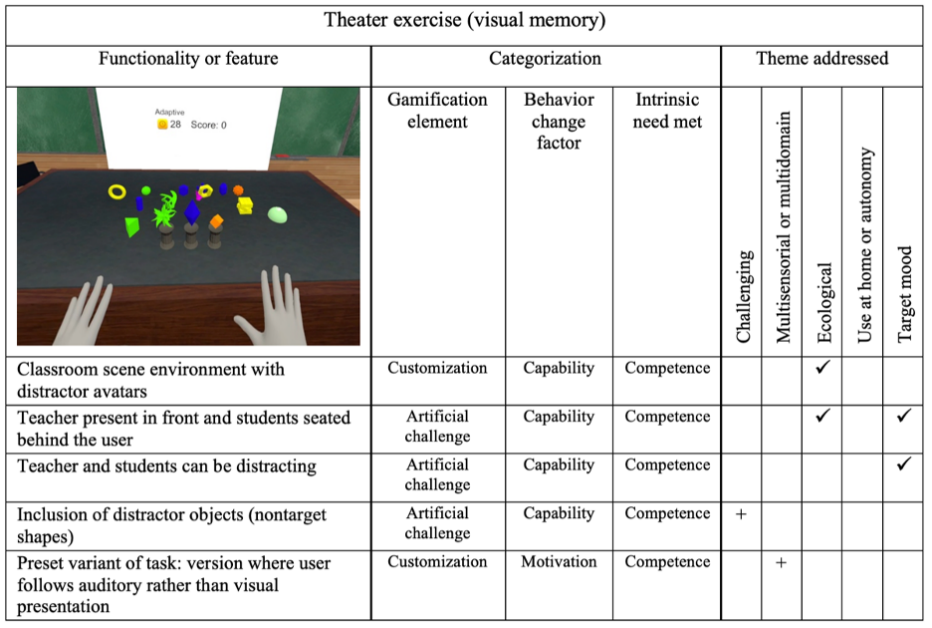
Theater exercise (targeting visual memory), including modifications made after end-user feedback. Checkmarks (✓) denote existing elements and plus signs (+) denote new elements that were added in the updated version of bWell-D.

Modifications to the scene and a summary of their categorization are presented in Figure 6, where it can be observed that the existing features did not include the emerging theme of multisensorial and multi-domain elements. A *customization* option was added to the scene in which a virtual voice gives instructions about which shape to place in which order instead of presenting the target shapes visually. Anticipating that this audio mode would be too difficult when the presented shapes were complex or unfamiliar, a new collection of number-shaped objects was created for use in the auditory mode.

### Tent Scene

The tent scene is intended as a space for relaxation and sensory immersion. The user is placed in a relaxing nature environment and allowed to freely explore. A ball that grows and shrinks to guide the user’s breathing, as well as additional user *customization* promoting relaxation, is included. Specifically, the user was given control of the selection of the environment and background music that plays. A virtual book placed in front of the user includes controls that allow them to navigate through a variety of virtual environments. Giving users control over their environment is expected to add motivation for continued use and address their need for autonomy.

**Figure 7:**
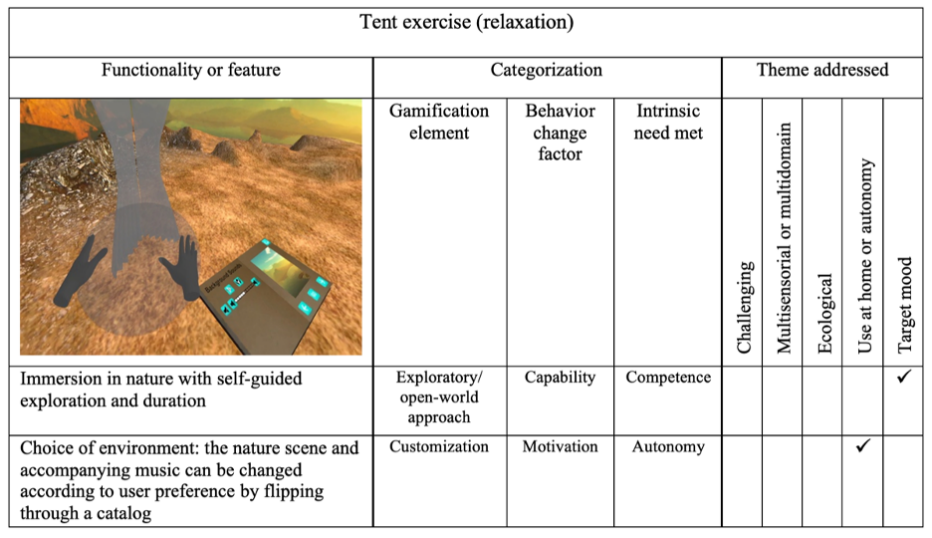
Tent exercise (targeting relaxation). A task to target mood was added to bWell-D based on end-user recommendations. This task was taken as is from the bWell platform core battery of exercises. Checkmarks (✓) denote existing elements.

While this scene was part of the bWell platform, it was not initially included in bWell-D. Feedback from end users about the need for activities that target mood prompted its inclusion. The scene was designed to follow an *exploratory* *open-world approach*, allowing users to freely look around the environment for as much or as little time as they wished. A summary of the features and their categorization is presented in Figure 7.

### Iterative Feedback

In the previously published qualitative study [53], a second round of interviews was conducted with 4 patients, during which they were shown the original bWell-D videos and updated videos that highlighted the added functionalities and features. All the participants reacted positively to these changes, felt that their input was considered, and thought that the initial version of bWell-D was improved.

In this study, the updated version was formally evaluated in participant trials with the original version in a single standardized session for initial assessment and as multi-session cognitive training for the improvements and gamification elements. In both the assessment and training sessions, the participants carried out the exercises for a total of 40 minutes. Of the 20 participants, 15 (75%) completed the assessment session, 4 (20%) opted to do the training sessions, and 1 (5%) tried the expanded features. The demographics and clinical characteristics of the participants of this study are summarized in Table 1 for reference. It was planned for each participant to interact with bWell for 1 assessment session, and those who opted in for training would have 8 more sessions. However, 25% (1/4) of the participants who elected to do the training sessions withdrew from the study after the second training session. User experience scales (GUESS and UES) were administered on training sessions 1, 4, and 8; therefore, 75% (3/4) of the participants rated the bWell-D expanded features 3 times, and 25% (1/4) of the participants rated bWell-D only once, for a total of 10 measures taken.

The results indicated that bWell-D was well tolerated by the tested participants in the initial and training sessions, with scores within the acceptable range in the literature (total SSQ score of <5 after the session) [57]. The SSQ scores, as summarized in Table 2, showed no significant increases (after vs before the session) in cybersickness symptoms in the initial assessment session or in the training sessions for the 20% (4/20) of the participants who opted in (the scores for the training sessions were measured as average SSQ score changes across all sessions). The GUESS, a 7-point Likert scale and whose results are summarized in Table 3, demonstrated high scores for user experience. In addition, for the improved version of bWell-D, an increase in score in 4 subscales (usability, enjoyment, audio aesthetics, and visual aesthetics) was observed. The effect size was large for usability and audio aesthetics, medium for visual aesthetics, and small for enjoyment [58]. While all subscales were higher for the improved bWell-D version, the small sample size and limited effect size do warrant further studies with larger populations. Although not statistically significant, engagement scores measured using the UES, a 5-point Likert scale with 4 subscales, remained high, with an average score of 15.07 (SD 1.53) for the initial session and 15.48 (SD 0.42) for the training sessions out of a possible maximum score of 20, as shown in Table 4.

Regarding the unstructured feedback during the training sessions, one participant mentioned that they did not feel the passage of time while playing the mole game and was surprised when their 10-minute game session was over. Another participant compared the mole game to the original arcade games they used to play years before, and their intense engagement with the game led to a noticeable perspiration and higher heartbeat levels. The theater game was mentioned as the most liked game by a participant, whereas a different person stated that they did not like the game in the initial session as it was hard to obtain a sense of achievement. One person specifically enjoyed the relaxation scene (tent), in particular the ability to freely explore the environment.

The participant that tried 1 session of bWell-D with expanded features stated that moving within the office space was interesting and led to a better experience compared to the original version of bWell-D. Regarding the laboratory scene, they reacted positively to the addition of ingredient mixing, believed that it would also make it more difficult, and made additional suggestions to further increase the complexity. In the mole game, they recognized the addition of faces to the moles and the multiple-hit moles as variants. Furthermore, they approved of the option to have moles coming from the sides more than an expanding table as it involves peripheral vision. However, they also mentioned that these elements should be introduced after practicing the initial task to avoid possible frustration. They also noted that the tent scene was indeed a relaxing environment.

**Table 1:** Characteristics of the nonclinical participants who took part in this study (N=20).

Participant characteristics	Values
Age (y), mean (SD)	41.8 (12.58)
Age (y), median (IQR)	43.5 (34.25-53.25)
Sex (female), n (%)	12 (60)
BDI^a^ (score range 0-63), mean (SD)	3.7 (5.55)
BDI (score range 0-63), median (IQR; SEM)	2 (0-4.25; 1.24)
STAI^b^, mean (SD)	59.85 (20.3)
STAI, median (IQR; SEM)	50.5 (46.50-68.25; 4.54)

^a^BDI: Beck Depression Inventory.

^b^STAI: State-Trait Anxiety Inventory.

**Table 2:** Scores for before and after the virtual reality session for the initial assessment and training sessions (Simulator Sickness Questionnaire [SSQ] scores averaged over all sessions).^a^

	Assessment session—original version			2-sided paired *t* test *P* value		Training sessions—improved version			2-sided paired *t* test *P* value
	Postsession SSQ score, mean (SD)	Presession SSQ score, mean (SD)			Postsession SSQ score, mean (SD)		Presession SSQ score, mean (SD)		
Total score	4.533 (9.672)	5.733 (13.014)	.14		1.550 (1.909)		2.125 (2.604)	.27	
General discomfort	0.200 (0.400)	0.200 (0.561)	>.99		0.050 (0.112)		0.100 (0.163)	.18	
Fatigue	0.267 (0.573)	0.533 (0.915)	.16		0.100 (0.105)		0.375 (0.468)	.18	
Boredom	0.133 (0.340)	0.400 (0.828)	.30		0.050 (0.112)		0.025 (0.056)	.37	
Drowsiness	0.067 (0.249)	0.333 (0.900)	.16		0.100 (0.163)		0.175 (0.274)	.21	
Headache	0.267 (0.680)	0.067 (0.258)	.19		0.000 (0.000)		0.025 (0.056)	.37	
Eyestrain	0.333 (0.596)	0.333 (0.617)	>.99		0.200 (0.381)		0.225 (0.437)	.37	
Difficulty focusing	0.200 (0.542)	0.333 (0.816)	.16		0.050 (0.112)		0.050 (0.068)	>.99	
Salivation increase	0.067 (0.249)	0.133 (0.516)	.33		0.000 (0.000)		0.025 (0.056)	.37	
Salivation decrease	0.067 (0.249)	0.067 (0.258)	>.99		0.000 (0.000)		0.025 (0.056)	.37	
Sweating	0.333 (0.471)	0.200 (0.561)	.43		0.225 (0.335)		0.125 (0.280)	.59	
Nausea	0.133 (0.340)	0.067 (0.258)	.33		0.000 (0.000)		0.000 (0.000)	>.99	
Difficulty concentrating	0.267 (0.573)	0.333 (0.816)	.33		0.025 (0.056)		0.050 (0.112)	.70	
Mental depression	0.067 (0.249)	0.200 (0.561)	.16		0.000 (0.000)		0.000 (0.000)	>.99	
“Fullness of the head”	0.267 (0.680)	0.267 (0.594)	>.99		0.000 (0.000)		0.025 (0.056)	.37	
Blurred vision	0.200 (0.542)	0.333 (0.724)	.16		0.225 (0.503)		0.250 (0.492)	.37	
Dizziness—eyes open	0.200 (0.542)	0.133 (0.516)	.33		0.075 (0.168)		0.050 (0.112)	.37	
Dizziness—eyes closed	0.067 (0.249)	0.200 (0.561)	.16		0.200 (0.447)		0.225 (0.503)	.37	
Vertigo	0.133 (0.499)	0.133 (0.516)	>.99		0.000 (0.000)		0.000 (0.000)	>.99	
Visual flashbacks	0.133 (0.499)	0.267 (0.799)	.16		0.000 (0.000)		0.000 (0.000)	>.99	
Faintness	0.067 (0.249)	0.133 (0.516)	.33		0.000 (0.000)		0.000 (0.000)	>.99	
Awareness of breathing	0.067 (0.249)	0.333 (0.816)	.10		0.000 (0.000)		0.000 (0.000)	>.99	
Stomach awareness	0.133 (0.499)	0.067 (0.258)	.33		0.100 (0.224)		0.200 (0.447)	.37	
Loss of appetite	0.133 (0.340)	0.200 (0.561)	.33		0.000 (0.000)		0.000 (0.000)	>.99	
Increased appetite	0.467 (0.718)	0.067 (0.258)	.05		0.000 (0.000)		0.050 (0.112)	.37	
Desire to move bowels	0.067 (0.249)	0.200 (0.561)	.33		0.100 (0.224)		0.100 (0.224)	>.99	
Confusion	0.133 (0.499)	0.200 (0.775)	.33		0.000 (0.000)		0.000 (0.000)	>.99	
Burping	0.067 (0.249)	0.000 (0.000)	.33		0.000 (0.000)		0.000 (0.000)	>.99	
Vomiting	0.000 (0.000)	0.000 (0.000)	>.99		0.000 (0.000)		0.000 (0.000)	>.99	
Other	0.000 (0.000)	0.000 (0.000)	>.99		0.050 (0.112)		0.025 (0.056)	.37	

^a^Total SSQ scores were computed based on the study by Bouchard et al [59]. Comparing post- versus presession scores, it was observed that there were no significant increases in SSQ scores in the initial session (15 participants) and the training sessions (4 participants).

**Table 3:** Comparison of Game User Experience Satisfaction Scale results across 15 participants for the assessment session (original version) and across 4 participants who opted for the training sessions (improved version), averaging 3 recordings per participant.^a^

	Usability/playability	Play engrossment	Enjoyment	Audio aesthetics	Personal gratification	Visual aesthetics
Assessment session (original version), mean (SD)	5.399 (0.573)	4.925 (0.887)	5.363 (1.050)	4.783 (1.372)	5.544 (0.717)	5.422 (1.080)
Multiple training sessions (improved version), mean (SD)	6.053 (0.500)	4.965 (0.647)	5.583 (0.514)	5.708 (0.241)	5.528 (0.718)	6.028 (0.048)
Effect size (Cohen *d*)	1.216	0.052	0.266	0.939	−0.022	0.793

^a^All scores were, on average, higher in the training sessions than in the assessment session. This trend of high training means relative to assessment means is captured in the effect size, with the *usability/playability* and *audio aesthetics* subscales having large effect sizes (<0.80) [58].

**Table 4:** Comparison of User Engagement Scale results across 15 participants for the assessment session (original version) and across 4 participants who opted for the training sessions (improved version), averaging 3 recordings per participant.^a^

	Focused attention subscale	Perceived usability subscale	Esthetic appeal subscale	Reward factor subscale	Overall engagement score (out of 20)
Assessment session (original version), mean (SD)	3.486 (0.438)	3.667 (0.452)	3.907 (0.696)	4.007 (0.465)	15.07 (1.526)
Training sessions (improved version), mean (SD)	3.345 (0.238)	3.938 (0.172)	4.200 (0.452)	4.000 (0.141)	15.483 (0.415)

^a^High engagement was observed for all sessions (overall engagement score of >15 out of 20).

## Discussion

### Principal Findings

#### Overview

In this paper, we propose a 2-pronged approach using end-user feedback for the co-design of content as well as in the application of gamification. This process considers behavior change theories to structure feedback obtained and provide insights in terms of drivers that potentially promote engagement and enhance intervention efficacy. Ultimately, the aim was to improve the compliance rate of a VR cognitive remediation intervention for depression.

Several interesting findings emerged with implications for technology designers involved in developing interventions for people with depression, as well as for the implementation of such interventions. Optimizing engagement is critical for the success of digital interventions for any patient cohort. However, it is an especially vital consideration for interventions targeting depression, which is defined by decreased ability to experience enjoyment or interest, decreased motivation, and a lack of self-efficacy [6]. Grounded in feedback obtained from persons with lived experience, this analysis provides useful guidance for developing intrinsically engaging, motivating digital interventions that can overcome these hallmark features of depression.

#### Identification of Gamification Elements

The classification of the gamification elements added to bWell-D indicated that feedback from end users centered on using gamification to add *personalization* and *customization* options, *artificial* *challenges*, and *artificial* *assistance*. In defining *customization* versus *personalization* elements, the former consist of settings controlled by the clinician or user, whereas the latter are settings automatically imposed by bWell-D. In defining *personalization* versus *artificial* *challenge* elements, the former are adaptations applied by the software in response to user characteristics and performance, whereas the latter are applied regardless of the user. Inclusion of these elements indicates that users expressed a need for challenges, tasks, and settings that are generic, as well as challenges and options that either are automatically tailored to the individual or the individual themselves chooses.

In an example of *customization*, the tent scene provides a variety of nature scenes that the user can choose from. It is worthwhile to note that, although it may seem interesting to develop additional virtual environments to reach more user-specific contexts, this requires careful consideration. For instance, one specific piece of feedback received from participants was that, although the office environment was created to represent a real-world setting, it still did not look like their workplace. Rather than creating a vast catalog of representative workplace settings, it was instead decided to include *bridge exercises* to provide the opportunity for the user to reflect on their specific context. Hence, it may be good practice to identify and consider the processes for which the intervention is intended to work and identify different potential options that can be implemented to support these processes. This would be of particular benefit in instances requiring substantial developmental effort.

Looking at the types of gamification elements included overall, while *personalization* and *customization* are among the most commonly observed gamification elements [48,49], the feedback obtained from end users also resulted in the inclusion of gamification elements in bWell-D that are less commonly observed, such as *artificial* *assistance*, *exploratory open-world approaches*, and *artificial* *challenge*.

#### COM-B Model Insights

On the basis of the results of the classification in terms of the driver of behavior according to the COM-B model, it is clear that the feedback received from our clinical collaborators focused on implementing features related to *capability* (19/28, 68% of the features). Interestingly, most of the gamification elements that were implemented centered on adding variety to the VR experiences (choice of environment, difficulty level adjustment, and types of stimuli and distractors). These features were implemented so that they can be turned on or off to promote engagement over time to enable new and more challenging features and game modes as the participant progresses throughout the intervention. This keeps the experience fresh, with the goal to help users stay the course of the remediation program. Evidence in support of this goal was provided by the UES results, where it was observed that participants maintained a high user engagement throughout the multiple sessions of the 8-week training program. Another advantage is that bWell-D offers a great deal of flexibility, permitting clinicians to adjust the configuration to the specific needs of different and potentially vulnerable populations. On that matter, Koivisto and Malik [60] present the importance of developing features that target a specific population and note that very few gamification studies focus on needs specific to older adults.

The elements that focused on *motivation* generally related to providing exercise variants based on the identified theme of leveraging the multisensorial affordances of VR for more fun and engaging experiences. For example, audio versions of the tasks were included as an alternative to visual presentation of instructions. It is likely that such elements contributed to the increase in GUESS scores for *usability/playability* and *audio and visual aesthetics* in the improved version of bWell-D.

Of the modifications to bWell-D, only two related to the *opportunity* aspect of the COM-B model: (1) providing the option for use at home and (2) inclusion of tutorials. From the feedback received, it was made clear that compliance and adherence depend not only on user engagement with the exercises but also on other factors such as ease of use and accessibility. Making bWell-D available for at-home use and accompanying it with tutorials in support has the potential to increase compliance by reducing the barriers to access it. Users can perform the intervention from the comfort of their own home and on their own schedule rather than having to travel to a clinic at a specific appointment time. The improved access is especially important for people with depression, who may find it *difficult to get out of bed*, and also extends the reach to diverse patient populations (eg, older adults with decreased mobility and those in remote or rural settings). While the inclusion of tutorials is a form of *artificial* *assistance*, providing the option for use at home was not classified as an element of gamification. It was observed that the typical gamification elements that target *opportunity* (cues and reminders or social mechanics to prompt users) were not included in the modifications. While the application of such gamification elements can be readily integrated into mobile interventions, in the case of a VR intervention, this is less straightforward. However, future iterations of bWell-D development will nonetheless aim to incorporate these. A potential and interesting avenue could be the development of a companion mobile app to prompt users at opportune moments by sending notifications.

#### Promoting Intrinsic Engagement

Efforts were intentionally made to choose modifications to bWell-D that enhance intrinsic motivation. Of the changes implemented, only 1 did not address the 3 fundamental needs outlined in self-determination theory, which are essential for psychological growth. This exception was the score streak indicator in the mole scene, which offers extrinsic motivation. Of the modifications made, most addressed the need for *competence*, which is not surprising given the context of cognitive remediation. Many of these modifications were related to increasing user engagement through the introduction of new features, adding multisensorial elements, or varying the difficulty of the exercise. The need for *autonomy* was addressed by 4 of the modifications. These were primarily related to providing user-selectable options and menus for customization and enabling at-home use. In total, bWell-D offers 3 types of gamified menus that users can access in different ways: by clicking on option tabs, by viewing their wrist in the game, and by flipping through the pages of a book provided in the scene. Each menu provides different types of information to the user.

Analysis of the results indicated that none of the modifications addressed the third need according to self-determination theory—*social* *connection*. Currently, social elements are only implemented in the exercises via nonplayable characters. From the taxonomy, it was observed that there are quite a few gamification elements related to social connection, specifically *social* *comparison*, *social* *competition*, *social* *cooperation*, and *social networking*. Given that the integration of social mechanics favors the *opportunity* domain of the COM-B model and that self-determination theory states that a sense of connection with others promotes self-actualization, this aspect will be prioritized in the next iterations of bWell-D. However, feedback from end users and domain experts is required to identify which of these elements are most appropriate for mental health domains.

### Limitations and Next Steps

A limitation in our previously published qualitative study is that, due to COVID-19–related restrictions, users who provided feedback did not experience the VR implementation firsthand as originally intended but instead watched videos of the implementation. This limited the type of feedback that the end users could provide on aspects such as game mechanics and usability. This study addressed these limitations by recruiting healthy participants to trial both the original and improved versions of bWell-D. Both structured and unstructured feedback was collected to evaluate the impact of the gamification elements. Another limitation is that the implemented features have not yet been tested on a clinical population. Therefore, it is not currently possible to evaluate whether the changes to our software have the expected impact on user engagement or intervention efficacy in this clinical population. We plan to obtain this feedback and measure adherence in upcoming 8-week bWell-D remediation trials with people with MDD. It is hoped that the modifications based on end-user feedback will lead to good compliance with the program. An additional limitation is the relatively small sample size of participants who completed the initial user trials and provided feedback on the optimized features of bWell-D.

Current literature exposes possibilities for gamification that are yet to be explored and evaluated. For instance, *randomness*, *artificial challenge*, *artificial assistance*, *exploratory or open-world approaches*, and *social* *cooperation* are reported to be underused for applications in the mental health domain, and further research is required to determine how these elements can best be used, if at all [49]. The mechanisms through which gamification impacts mental health in the real world and which gamification elements are associated with more benefits should also be further explored. bWell-D has integrated a number of these underused elements based on the iterative feedback obtained, and with the iterative development process in place, it will be feasible to take on such investigations.

### Conclusions

The bWell-D prototype was developed following best practices in VR clinical application design using iterative feedback from both patients and health care providers, resulting in the development of a VR cognitive remediation tool that was demonstrated to be acceptable and provided a favorable user experience with sustained engagement. We presented a process for the application of gamification that includes characterizing modifications in a standardized way and identifying the underlying mechanisms that are targeted. This classification revealed that the gamification elements implemented in the prototype included some of those less commonly observed in other applications and tended to address intrinsic motivations. In addition, the modifications made were found to address all 3 drivers of behavior identified in the COM-B model. As such, the latest iteration of bWell-D appears to be well poised to promote user uptake and compliance. In future developments, we plan to incorporate elements that are currently missing to ensure a balanced representation of the factors identified from behavioral theories.

## Appendix

Multimedia Appendix 1 Video of the bWell interface and laboratory scene of the original prototype version.

## Abbreviations

CCN: cognitive care network
COM-B: capability, opportunity, and motivation–behavior
GUESS: Game User Experience Satisfaction Scale
MDD: major depressive disorder
NRC: National Research Council Canada
SSQ: Simulator Sickness Questionnaire
UES: User Engagement Scale
VR: virtual reality
